# Forsythoside A Alleviates Neuroinflammatory Damage via Inhibiting TLR4/NF‐κB/NLRP3 Activation‐Induced Astrocyte Pyroptosis in Cerebral Ischemia–Reperfusion

**DOI:** 10.1002/cns.70867

**Published:** 2026-04-08

**Authors:** Yupeng Guo, Xuanwei Dong, Min Liu, Jianxin Wang, Shewei Guo

**Affiliations:** ^1^ Department of Neurosurgery Aviation General Hospital Beijing China; ^2^ Neurosurgical Department The First Affiliated Hospital of Zhengzhou University Zhengzhou Henan China

**Keywords:** cerebral ischemia reperfusion, forsythoside a, NLRP3 inflammasome, pyroptosis, TLR4/NF‐κB pathway

## Abstract

**Background:**

The neuroinflammatory cascade triggered by cerebral ischemia–reperfusion injury (CIRI) represents a pivotal pathological mechanism driving neuronal death and dysfunction. Forsythoside A (FA) exhibits anti‐inflammatory properties, but its effects on CIRI and potential mechanisms remain unclear.

**Aims:**

To investigate whether FA improves astrocyte pyroptosis and alleviates neuroinflammatory damage in CIRI via modulating the Toll‐like receptor 4 (TLR4)/nuclear factor κB (NF‐κB)/NLRP3 pathway.

**Methods:**

The middle cerebral artery occlusion reperfusion (MCAO/R) mouse model was established, with cerebral infarction volume observed through TTC staining, and neurological scores assessed. The effects of FA on cerebral cortical damage, neuroinflammation, apoptosis, and pyroptosis were observed by pathological staining. An oxygen–glucose deprivation/reoxygenation (OGD/R) U251 human astroglioma cell model was established, and the protective impact of FA on cell viability was detected through the cell counting kit‐8 assay. The release of lactate dehydrogenase (LDH) was measured by the LDH assay Kit, and cell pyroptosis was evaluated through Hoechst 33,342/PI staining. Inflammatory factor levels were detected by ELISA kits, with pyroptosis‐related and TLR4/NF‐κB/NLRP3 pathway‐related protein levels evaluated through Western blot.

**Results:**

FA reduced the infarct volume, ameliorated brain tissue pathological damage, reduced activated astrocytes, and inhibited neuronal apoptosis in MCAO/R mice. FA also alleviated inflammatory cell infiltration in brain tissue and declined pro‐inflammatory factor levels in serum. In addition, FA suppressed TLR4 and p‐NF‐κB p65 expression and NLRP3 inflammasome activation in astrocytes. OGD/R caused decreased U251 cell viability, increased LDH release, and pyroptosis rate, while FA treatment could reverse the above effects. Mechanistically, FA hindered the TLR4/NF‐κB pathway, down‐regulated NLRP3 and pyroptosis‐related proteins. The TLR4 agonist LPS weakened the protective impact of FA on MCAO/R mice and OGD/R cell models.

**Conclusion:**

FA alleviates neuroinflammatory damage through inhibiting the TLR4/NF‐κB axis and blocking NLRP3 inflammasome‐induced astrocyte pyroptosis.

## Graphical abstract

Forsythoside A exerts a neuroprotective effect by inhibiting the activation of the TLR4/NF‐κB signaling axis, thereby blocking the assembly and activation of the NLRP3 inflammasome and further suppressing astrocytic pyroptosis and the release of inflammatory cytokines.

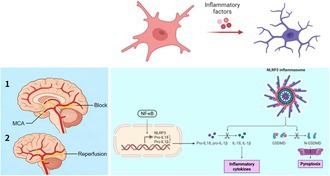

**Abbreviations**
BBB: blood–brain barrier
BSA: Bovine serum albumin
CCK‐8: Cell Counting Kit‐8
CIRI: cerebral ischemia–reperfusion injury
FA: Forsythoside A
GFAP: Glial Fibrillary Acidic Protein
HE: Hematoxylin and eosin
IL: Interleukin
iNOS: inducible nitric oxide synthase
LDH: lactate dehydrogenase
MCAO/R: Middle cerebral artery occlusion reperfusion
MyD88: Myeloid differentiation factor‐88
NF‐κB: nuclear factor‐kappaB
Nig: nigericin
NLRP3: NOD‐like receptor protein 3
OGD/R: Oxygen–glucose deprivation/reoxygenation
PI: Prodium Iodide
TLR4: Toll‐like receptor 4
TNF‐α: tumor necrosis factor‐α
TUNEL: TdT‐mediated dUTP Nick‐End Labeling

## 1 Introduction

As the social economy progresses, the aging of the population is becoming increasingly serious, and the incidence of cardiovascular and cerebrovascular diseases continues to rise [1, 2]. According to statistics, stroke ranks as the second primary cause of death, with ischemic stroke representing approximately 85% of all occurrences [3]. Currently, the only medication approved for the restoration of blood flow is recombinant tissue plasminogen activator, but its narrow therapeutic time window limits its application, leading to elevated disability and death rates in ischemic stroke [4, 5]. The key pathological mechanism of ischemic stroke is cerebral ischemia–reperfusion injury (CIRI), which involves oxidative stress, the blood–brain barrier (BBB) disruption, and neuroinflammation [6, 7]. When blood flow is restored to the ischemic brain tissue, immune cell infiltration, the release of inflammatory mediators, and abnormal activation of glial cells lead to secondary brain damage, causing neurological deficits and neuronal death [8]. Excessive activation of astrocytes in CIRI not only exacerbates the inflammatory response by releasing proinflammatory factors, but also further amplifies neuroinflammation through pyroptosis [9, 10]. Therefore, inhibiting astrocyte pyroptosis and thus improving neuroinflammation may be an effective way to improve the CIRI prognosis.

Numerous investigations have found that natural active ingredients have the characteristics of few side effects, low toxicity, variable bioavailability, complex chemical structure, and diverse biological activities, showing promise in treating various diseases [11, 12]. Derived from the fruit of the traditional Chinese medicinal plant *Forsythia suspensa* , Forsythiaside A (FA) is a phenylethanol glycoside compound, recognized for its multiple pharmacological benefits, like anti‐inflammatory, neuroprotective, and antioxidant [13, 14, 15]. Recent investigations reveal that FA exhibits significant protective impacts in a variety of neurological disease models [15, 16]. In the Alzheimer's disease model, FA can inhibit amyloid‐β deposition, reduce neuronal ferroptosis and neuroinflammation, and improve cognitive dysfunction [15]. In the ischemic stroke model, FA reduces oxidative stress and promotes neurological function recovery via stimulating the Nrf2/HO‐1 pathway [16]. However, the way FA protects against CIRI‐induced astrocyte pyroptosis and neuroinflammation is still unclear.

Toll‐like receptor 4 (TLR4) serves as a crucial pattern recognition receptor that mediates inflammatory responses in CIRI. After activation, it can recruit downstream molecules via myeloid differentiation factor 88 (MyD88), thereby causing nuclear factor κB (NF‐κB) phosphorylation [17]. Activated NF‐κB upregulated inflammatory genes like interleukin (IL)‐1β and NOD‐like receptor protein 3 (NLRP3), further promoting neuroinflammation [18, 19]. A study has confirmed that overactivation of the TLR4/NF‐κB/NLRP3 axis in astrocytes is a key driver of pyroptosis [20]. In addition, in a CIRI mouse model caused by middle cerebral artery occlusion reperfusion (MCAO/R), interferon‐inducible protein 35 can induce upregulation of TLR4 expression, thereby activating NF‐κB and encouraging the assembly of NLRP3 inflammasomes, ultimately exacerbating neuroinflammation [21]. Therefore, blocking the TLR4/NF‐κB/NLRP3 signaling axis may be an effective strategy to inhibit astrocyte pyroptosis and reduce neuroinflammation. Herein, we developed a cell model of oxygen–glucose deprivation/reoxygenation (OGD/R), along with a mouse model of MCAO/R, to investigate whether FA inhibits astrocyte pyroptosis and neuroinflammation via blocking the TLR4/NF‐κB/NLRP3 pathway. This work is intended to clarify the specific mechanism by which FA mitigates CIRI and provides new references for the clinical utilization of FA and the drug development of CIRI.

## 2 Materials and Methods

### 2.1 Construction of the MCAO/R Mouse Model

Male C57BL/6 mice (3 months old, 21–24 g) were purchased from Vitalriver (Beijing, China) and housed under constant temperature conditions of 22°C, with humidity maintained at 45%–50%, a 12 h/12 h light–dark cycle. Mice were assigned randomly to a sham group (*n* = 10) and an experimental group (*n* = 50). After anesthetizing using 2% isoflurane, the surgical area of the mice was thoroughly disinfected with 75% ethanol and 10% iodine. In the experimental group, nylon thread was placed at the distal common carotid artery (CCA) and proximal ends of CCA, and the external carotid artery (ECA), and the CCA and ECA were ligated at the proximal end, respectively [22]. Then, a small incision was created 4 mm from the CCA bifurcation, and the ligature was placed into the internal carotid artery (ICA). Using ophthalmic forceps, the ligature was carefully advanced. Upon reaching a depth of approximately 20 mm and encountering resistance, the ligatures at the distal ends of ICA and CCA were tied to fix the ligature. The incision was sutured layer by layer and disinfected, and a suture of appropriate length was left outside the body for reperfusion. Following a 1.5‐h interruption of blood circulation [22], the suture was removed, and reperfusion was performed. It is crucial to maintain the body temperature of the mice throughout the entire process. Mice in the sham group were sutured and disinfected, but did not undergo MCAO/R surgery. The experimental group mice were divided randomly into five groups and injected intraperitoneally with normal saline, FA (20, 30, 60 mg/kg, HY‐N0028, MedChemExpress, Monmouth Junction, NJ, USA), and FA (60 mg/kg) + TLR4 agonist lipopolysaccharide (LPS, 10 mg/kg, L5293, Sigma‐Aldrich, St. Louis, MO, USA) 1 h after MCAO/R, respectively [23, 24]. They were designated as MCAO/R, FA‐L, FA‐M, FA‐H, and FA‐H + TLR4 groups, with 10 mice in each group.

At 24 h post‐MCAO/R surgery, neurological impairment in mice was assessed according to the neurological deficit score (mNSS) [25]. The mNSS score is on a scale of 0 to 14, with normal status being 0 and maximum neurologic deficit being 14. A total score of 10 to 14 indicates severe neurologic deficit, 5 to 9 indicates moderate neurologic deficit, and 1 to 4 indicates mild neurologic deficit. All mNSS tests were completed by independent examiners who were unaware of the grouping information. After the mNSS test was completed, mice were euthanized with sodium pentobarbital (150 mg/kg), blood was collected, and the brain tissues were then harvested and quickly stored in a −80°C refrigerator.

### 2.2 Triphenyltetrazolium Chloride (TTC) Staining

The brain tissue underwent freezing at −80°C for 15 min. After freezing, it was placed into a pre‐prepared fixed mold and sliced into a 2 mm thickness. Subsequently, the tissue sections were exposed to TTC solution (2%, C0652, Beyotime, Shanghai, China) preheated to 37°C and incubated in a dark environment at 37°C for 15 min. At approximately 7 min, the brain tissue was flipped to ensure uniform and adequate staining. After incubation, photographs were taken, and the cerebral infarction volume was evaluated using ImageJ 1.54 h software (Wayne Rasband, National Institute of Mental Health, Bethesda, MD, USA).

### 2.3 Hematoxylin and Eosin (HE) Staining

Brain tissues of mice were immersed in 4% paraformaldehyde (158,127, Sigma‐Aldrich), paraffin‐embedded, and serially sectioned 48 h later. The sections were dried and deparaffinized with xylene (534,056, Sigma‐Aldrich), and then rehydrated using gradient ethanol. Following a 10‐min staining with hematoxylin solution (H810910, Macklin, Shanghai, China), the sections were rinsed and exposed to differentiation solution (A769718, Macklin) for 30 s. After thorough washing, sections were dyed with 1% eosin (C0109, Beyotime) for 60 s, dehydrated, transparentized using xylene, subsequently covered with Neutral Balsam (C0173, Beyotime) and observed with a DM IL LED microscope (Leica, Heidelberg, Germany). Using ImageJ 1.54 h software, cell counts were performed for each field of view, and the number of damaged cells (cells with nuclear consolidation and nuclear fragmentation) was counted separately from the total number of cells. The percentage of damaged cells = (number of damaged cells/total number of cells) × 100%.

### 2.4 Nissl Staining

The paraffin sections were spread flat in warm water and fully unfolded. After the tissue was stretched, they were picked up with a slide. The sections were placed in a drying oven (65°C) for 90 min, deparaffinized with xylene for 20 min, and rehydrated in gradient alcohol. The liquid on the sections was aspirated, then Nissl staining solution (C0117, Beyotime) was added to cover the samples, and incubated for 10 min at 25°C. Following being fully rinsed with PBS, samples were placed in ethanol (95%) for dehydration for 4 min, and then transparentized in xylene. After sealing using Neutral Balsam, the samples were observed through a microscope. Five non‐overlapping fields of view were randomly selected, and the number of Nissl‐positive neurons in each field of view was counted using ImageJ 1.54 h software.

### 2.5 Construction of OGD/R Cell Model

Human astroglioma cells U251 were sourced from Wuhan Sunncell Biotechnology (Hubei, China) and grown in a U251 cell‐specific culture medium (SNLM‐095, Wuhan Sunncell Biotechnology) at 37°C with 5% CO_2_.

After U251 cells were routinely cultured for 24 h, they were transferred to a glucose‐free and serum‐free basal medium (PM150270, Wuhan Pricella Biotechnology, Hubei, China) and cultured in an anoxic incubator (95% N_2_, 5% CO_2_) for 4 h. Subsequently, cells were transferred to regular culture medium and placed in a 5% CO_2_ incubator for 24 h to construct the OGD/R cell model [26, 27]. In addition, for the FA group, U251 cells were exposed to FA (5, 10, 20, 40, 80, 100, 120 μM) for 3 h before the OGD/R cell model was constructed [15]. For the FA + NLRP3 agonist nigericin (Nig) group, U251 cells were first exposed to FA (80 μM) for 3 h, and the OGD/R cell model was constructed in the culture medium supplemented with Nig (10 μM, HY‐127019, MedChemExpress) [28]. For the FA + LPS group, U251 cells were first exposed to FA (80 μM) for 3 h, followed by OGD/R model construction in cell culture medium including LPS (100 ng/mL) [29].

### 2.6 Cell Counting Kit‐8 (CCK‐8) Assay

U251 cells were plated in 96‐well plates at 1 × 10^4^ cells per well (100 μL). When the cells were completely attached (approximately 12–16 h), varying concentrations of FA were introduced and cultured for another 24 h. Subsequently, 10% CCK‐8 reagent (HY‐K0301, MedChemExpress) was introduced, mixed thoroughly with the cells, and cultured for another 2 h. The OD_450_ value was examined through a SpectraMax Mini microplate reader (Molecular Devices, San Jose, CA, USA).

### 2.7 Hoechst 33,342 and Propidium Iodide (PI) Staining

Referring to Wang et al. [30], pyroptosis in U251 cells was assessed using Hoechst 33,342 staining and PI staining. U251 cells were introduced into 12‐well plates (coverslips pre‐placed in the wells). After different treatments, the cell slides were immersed in 4% paraformaldehyde for half an hour. Hoechst 33,342 staining solution (C1025, Beyotime) was introduced and cultured for half an hour at 4°C. The liquid was discarded, and cells were viewed using a fluorescence microscope. Additionally, the cells were incubated with 20 μg/mL of PI staining solution (ST511, Beyotime) in the dark for half an hour at 25°C, washed, and observed using a fluorescence microscope.

### 2.8 Lactate Dehydrogenase (LDH) Assay

The LDH Assay Kit (ab102526, Abcam, Cambridge, MA, USA) served to evaluate the membrane integrity of U251 cells [31]. U251 cells were introduced into a 96‐well plate at an appropriate density. When the cell confluence reached about 70%, FA was added for 3 h, and then Nig was added, and OGD/R treatment was performed. The supernatant was gathered, mixed thoroughly with the LDH detection working solution at 25°C for 30 min in darkness; OD490 values were measured using a microplate reader.

### 2.9 Immunofluorescence and TdT‐Mediated dUTP Nick‐End Labeling (TUNEL) Staining

Mouse brain tissue paraffin sections underwent deparaffinization using xylene and were subsequently hydrated in gradient ethanol, and then underwent microwave antigen retrieval. 0.3% Triton X‐100 (T824275, Macklin) was dripped on the surface of the tissue sections for permeabilization for 10 min. After adding 5% bovine serum albumin (BSA, A801320, Macklin) for blocking for 1 h at 25°C, primary antibodies NLRP3 (ab270449, 1:50), myeloperoxidase (MPO, ab208670, 1:1000), Glial fibrillary acidic protein (GFAP, ab17260, 1:5000), GSDMD‐N (ab215203, 1:100), p‐NF‐κBp65 (ab131100, 1:100) from Abcam were dripped on the tissues and incubated for 12 h at 4°C. The following day, sections were exposed to goat anti‐rabbit IgG (ab150081, 1:100, Abcam) for 90 min. After rinsing with PBS, sections were covered with AntiFade Mounting Medium (HY‐K1047, MedChemExpress), and the development was observed using a microscope.

For U251 cells, they were inoculated into 12‐well plates (cover glass was already placed in the plate) and left to react for 24 h. Subsequently, the cell slides were treated with 4% paraformaldehyde for about 20 min and then exposed to 0.3% Triton X‐100 for 10 min, and the rest of the operations were consistent with the fluorescent staining steps for brain tissue sections.

In addition, referring to Li et al. [22], neuronal apoptosis was detected through a TUNEL staining kit (C1086, Beyotime). Brain tissue sections were incubated for 12 h with NeuN primary antibody (ab177487, 1:200, Abcam) at 4°C. Subsequently, the TUNEL detection solution was introduced to the surface of the sections, then incubated for 1.5 h in darkness. The stained area was assessed using ImageJ software to evaluate neuronal apoptosis.

### 2.10 Elisa

Mouse tumor necrosis factor‐α (TNF‐α, PT512), IL‐6 (PI326), IL‐1β (PI301), and IL‐18 (PI553) ELISA Kits, along with Human TNF‐α (PT518), IL‐6 (PI330), IL‐18 (PI558), and IL‐1β (PI305) ELISA Kits, were used to evaluate the levels of inflammatory factors. All these ELISA Kits were purchased from Beyotime. Serum, brain tissue homogenate supernatant, or U251 cell supernatant and corresponding antibodies were introduced to the ELISA plate, and left to incubate for 1.5 h at 37°C. Subsequently, HRP‐labeled Streptavidin was introduced and incubated for 20 min. Following washing with the wash buffer, color developer A and B were introduced and incubated for another 15 min. Lastly, the stop solution was mixed thoroughly with, and the OD450 values were examined using a microplate reader.

### 2.11 Western Blot

The mouse brain tissue was minced and added to RIPA lysis buffer (P0013B, Beyotime), and then ground thoroughly to break the cells. U251 cells were gently rinsed with pre‐cooled PBS and mixed with RIPA lysis buffer. After lysis, the protein levels were evaluated through the BCA Protein Quantification Kit (B917925, Macklin). Next, proteins were separated via SDS‐PAGE, transferred onto a polyvinylidene difluoride membrane (Invitrogen, Carlsbad, CA, USA), and covered with 5% BSA for 2 h. Following rinsing the membrane, it underwent an overnight incubation with primary antibodies IL‐1β (P420B, 1:500, Invitrogen), GSDMD (PA5‐116815, 1:500, Invitrogen), GSDMD‐N (ab215203, 1:1000, Abcam), TLR4 (48–2300, 1200, Invitrogen), Caspase‐1 (PA5‐87536, 1:2000, Invitrogen), NF‐κB‐p65 (ab76311, 1:5000, Abcam), p‐NF‐κB‐p65 (44‐711G, 1500, Invitrogen), NLRP3 (MA5‐32255, 1:500, Invitrogen), ASC (ab283684, 1:1000, Abcam), inducible nitric oxide synthase (iNOS, ab283655, 1:1000, Abcam), or Cl‐Caspase‐1 (HY‐P80622, 1:500, MedChemExpress) at 4°C. On the second day, the membrane was exposed to goat anti‐rabbit IgG (ab97051, 1:2000, Abcam) at 25°C for 2 h. ECL working solution (HY‐K1005, MedChemExpress) was prepared and evenly dropped on the membrane, which was then imaged using an iBright CL1500 gel imaging system (Invitrogen). The analysis of gray values was conducted with ImageJ software, with the ratio of gray value to GAPDH (ab128915, 1:10000, Abcam) representing the relative expression level.

### 2.12 Molecular Docking

The 3D structure files for the TLR4 receptor proteins were acquired from the PDB database (https://www.rcsb.org/). Concurrently, the 3D structure file for FA was obtained from the PubChem database (https://pubchem.ncbi.nlm.nih.gov/). Receptors and ligands were processed using PyMOL 2.5.0 (Schrödinger Inc., NY, USA) and AutoDock Tools 1.5.7 (Scripps Research, CA, USA), respectively, to generate pdbqt format files. The molecular docking of receptors and ligands was carried out using AutoDock Vina. Following this, the molecular docking data were brought into PyMOL 2.5.0 software to be visualized.

### 2.13 Scanning Electron Microscope (SEM)

U251 cells were mixed with 2.5% electron microscopy fixative (G708101, Macklin) and fixed at 4°C away from light for 4 h. The cells were washed twice with PBS to remove the fixative and then dehydrated with gradient ethanol [32]. The cells were treated with isoamyl acetate (79,857, Sigma‐Aldrich) twice, each time for 10 min. After centrifugation, the cell precipitates were mixed with a small amount of PBS and added dropwise to the silicon wafers and dried at 37°C. Gold was sprayed for 150 s and finally observed and photographed by SEM (S‐4800, Hitachi, Tokyo, Japan).

### 2.14 Statistical Analysis

Each experiment underwent a minimum of three repetitions, with the results reported as mean ± standard deviation. SPSS 26.0 software (IBM SPSS Statistics 26) was used for statistical analysis. When conducting between‐group analyses, comparisons of the means of two independent samples were performed through an unpaired *t*‐test. A test of homogeneity of variances was routinely performed, and if the variances were not homogeneous, the analysis was repeated using an approximate *t*‐test. One‐way ANOVA was utilized to conduct mean comparisons across several groups. When variances were homogeneous, multiple comparisons between groups were carried out with Tukey's test; Dunnett's T3 test was used when variances were heterogeneous. *p* < 0.05 indicated a significant difference. Prism software (Graphpad 9.0) was utilized for drawing.

## 3 Results

### 3.1 FA Declined Infarct Volume and Brain Tissue Pathological Damage in MCAO/R Mice

**FIGURE 1:**
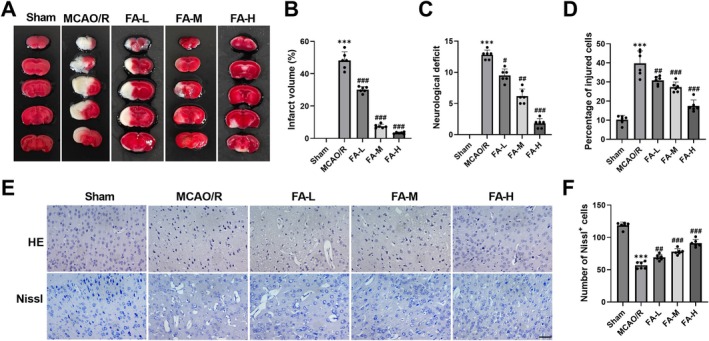
FA reduces cerebral infarction volume and improves pathological damage to brain tissue. (A–B) Clear infarct lesions (white areas) were observed in MCAO/R mice through TTC staining, whereas FA treatment declined infarct volume. (C) In MCAO/R mice, FA treatment dose‐dependently reduced mNSS scores. (D–F) HE and Nissl staining showed that MCAO/R mice exhibited cortical damage accompanied by a decrease in Nissl‐positive cells. FA treatment lowered the extent of brain tissue damage and increased Nissl‐positive cells (40×, 50 μm). ***p < 0.001 vs. Sham; *p < 0.05, **p < 0.01, ***p < 0.001 vs. MCAO/R.

TTC staining showed that MCAO/R model mice had clearly bordered white infarct foci in the cerebral cortex. FA treatment decreased the volume of cerebral infarction in a manner dependent on dosage, suggesting that FA can inhibit the process of tissue necrosis after cerebral ischemia (Figure 1A,B). The mNSS score results showed that MCAO/R model mice exhibited obvious neurological dysfunction, and FA intervention reduced the neurological deficit scores of mice (Figure 1C). The MCAO/R model group exhibited typical ischemic damage in the cerebral cortex as observed through histopathological staining: HE staining showed disordered neuronal structure, nuclear condensation, and inflammatory cell infiltration; Nissl staining further confirmed that Nissl bodies in the ischemic area were markedly reduced. FA treatment alleviated the pathological manifestations of brain tissue, the cell arrangement tended to be regular, the phenomenon of neuronal nuclear condensation was reduced, and Nissl‐positive cells were notably increased (Figure 1D–F). These findings indicate that FA can reduce cerebral infarction volume, alleviate MCAO/R‐induced neurological dysfunction in mice, and protect the structural integrity of neurons.

### 3.2 Inhibitory Effects of FA on Neuroinflammation and Neuronal Apoptosis in the Ischemic Penumbra of MCAO/R Mice

**FIGURE 2:**
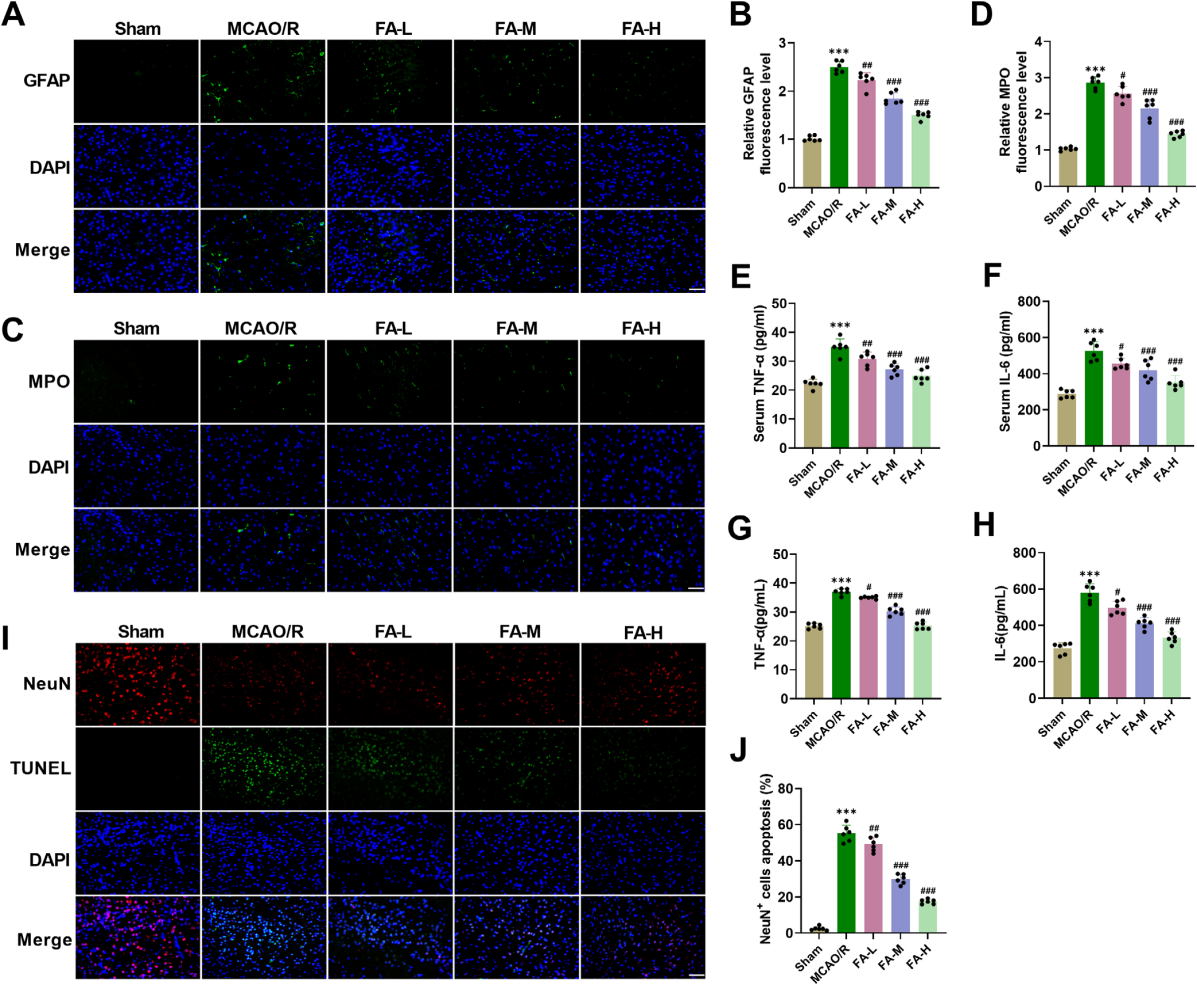
FA reduces neuroinflammatory damage and neuroapoptosis in the ischemic penumbra of MCAO/R mice. (A–B) Immunofluorescence showed that GFAP fluorescence intensity was heightened in the ischemic penumbra of MCAO/R mice, whereas FA treatment declined GFAP fluorescence intensity (40×, 50 μm). (C–D) After MCAO/R, MPO fluorescence intensity elevated in ischemic penumbra, while FA treatment reduced MPO fluorescence intensity (40×, 50 μm). (E–H) IL‐6 and TNF‐α levels in the serum (E, F) and brain tissue homogenate supernatant (G, H) of MCAO/R mice were detected using ELISA kits. (I–J) In MCAO/R mice, Immunofluorescence NeuN/TUNEL double staining indicated that the rate of neuronal apoptosis increased, and FA treatment reduced neuronal apoptosis (40×, 50 μm). ***p < 0.001 vs. Sham; *p < 0.05, **p < 0.01, ***p < 0.001 vs. MCAO/R.

Previous researches have shown that astrocytes, microglia, etc. are rapidly activated after reperfusion, releasing numerous pro‐inflammatory factors, triggering neuroinflammation [17, 33]. GFAP is a marker of astrocyte activation. In MCAO/R mice, immunofluorescence demonstrated that GFAP fluorescence intensity was markedly enhanced, indicating excessive activation of astrocytes in the ischemic penumbra. After FA intervention, the GFAP fluorescence intensity was decreased, indicating that it can suppress the abnormal activation of astrocytes (Figure 2A,B). After MCAO/R surgery, MPO fluorescence intensity, an inflammatory marker, was raised markedly in ischemic penumbra, and FA treatment reduced the fluorescence intensity of MPO, suggesting that it can inhibit neuroinflammation (Figure 2C,D). ELISA results showed that proinflammatory factors TNF‐α and IL‐6 levels in the serum (Figure 2E,F) and brain tissue homogenate supernatant (Figure 2G,H) of MCAO/R mice were markedly elevated, while FA treatment dose‐dependently reduced the concentrations of the above cytokines, further confirming that FA can inhibit inflammatory response. NeuN/TUNEL immunofluorescence co‐staining indicated that the proportion of TUNEL‐positive cells among NeuN‐positive neurons was notably elevated. After FA intervention, the neuronal apoptosis rate was markedly reduced (Figure 2I,J). These results confirm that FA can alleviate neuroinflammation and inhibit neuronal apoptosis, thereby improving MCAO/R‐induced neuronal damage.

### 3.3 FA Inhibits NLRP3 Inflammasome Activation and Pyroptosis in Astrocytes in MCAO/R Mice

**FIGURE 3:**
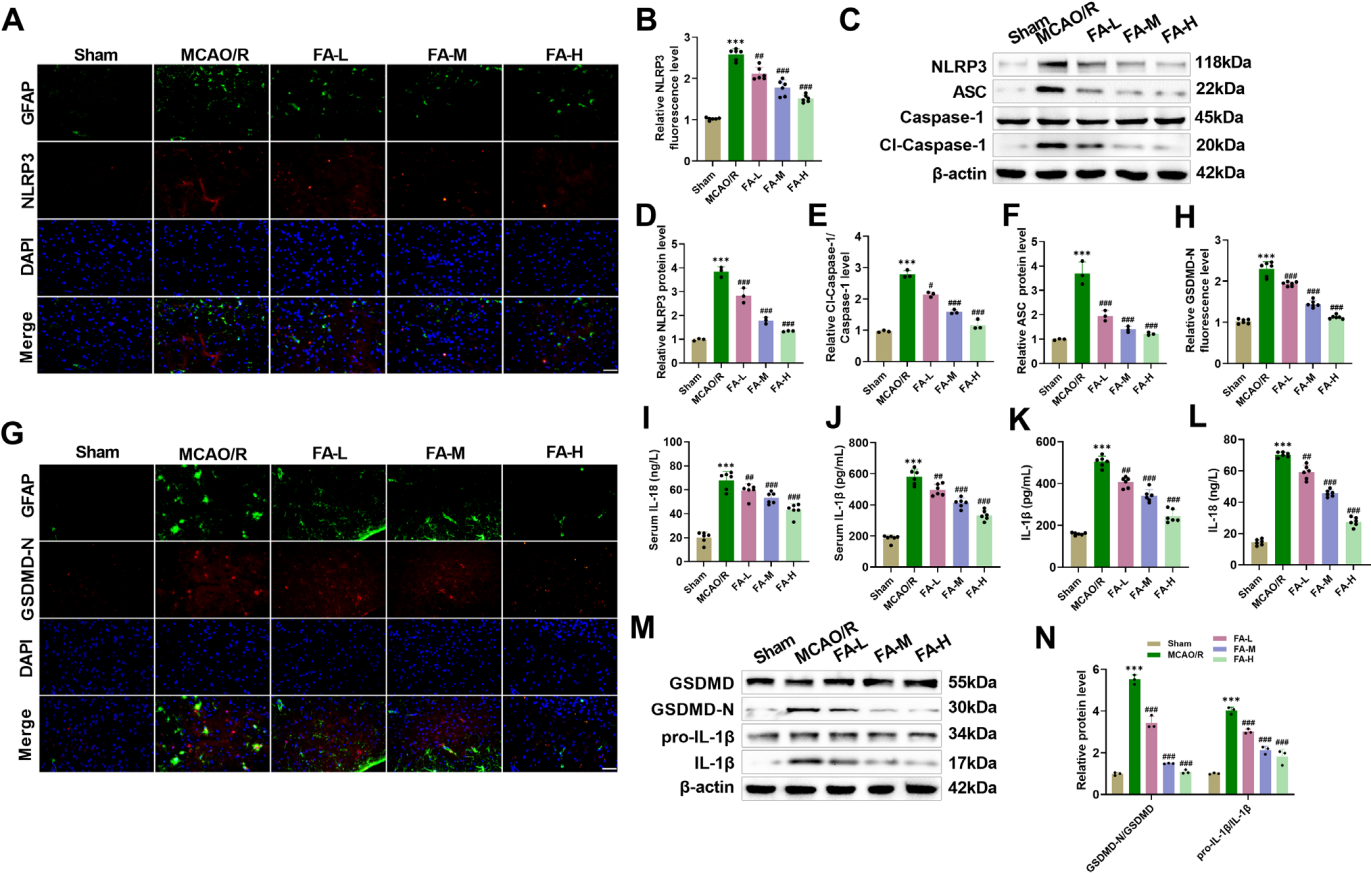
FA reduces NLRP3 expression and pyroptosis in CIRI‐induced astrocytes. (A–B) In MCAO/R mice, immunofluorescence showed colocalization of NLRP3 and GFAP in brain tissue, whereas FA treatment reduced the fluorescence intensity of NLRP3 (40×, 50 μm). (C–F) Elevated NLRP3, Cl‐Caspase‐1/Caspase‐1, and ASC levels in MCAO/R mice were measured by Western blot, while FA reduced these protein levels. (G–H) Immunofluorescence showed colocalization of GSDMD‐N and GFAP in MCAO/R mice, while FA reduced the fluorescence intensity of GSDMD‐N (40×, 50 μm). (I–L) IL‐1β and IL‐18 levels in the serum (I, J) and brain tissue homogenate supernatant (K, L) of MCAO/R mice were assessed through ELISA kits. (M–N) Elevated GSDMD‐N and IL‐1β levels in MCAO/R mice were measured by Western blot, whereas FA reduced the levels of both. ***p < 0.001 vs. Sham; *p < 0.05, **p < 0.01, ***p < 0.001 vs. MCAO/R.

To explore the specific mechanism through which FA alleviates pathological damage and neuroinflammation in MCAO/R mice, we detected the activation of NLRP3 inflammasomes in mouse brain tissue through immunofluorescence colocalization. In MCAO/R mice, NLRP3 and astrocyte marker GFAP showed significant colocalization in the ischemic penumbra tissue, suggesting that NLRP3 inflammasomes were mainly expressed in activated astrocytes. After FA intervention, NLRP3 fluorescence intensity was decreased dose‐dependently (Figure 3A,B). NLRP3, ASC, and Cl‐Caspase‐1/Caspase‐1 levels in MCAO/R mice were notably increased, as revealed by Western blot. FA treatment downregulated the above proteins, indicating that FA suppressed inflammasome activation (Figure 3C–F). In addition, in the MCAO/R group, GSDMD‐N and GFAP also showed obvious colocalization in ischemic penumbra, indicating the activation of astrocyte pyroptosis. After FA intervention, the fluorescence intensity of GSDMD‐N was significantly weakened, confirming that it can inhibit astrocyte pyroptosis (Figure 3G,H). Pyroptosis‐characteristic cytokines IL‐18 and IL‐1β levels in the serum (Figure 3I,J) and brain tissue homogenate supernatant (Figure 3K,L) of MCAO/R mice were markedly elevated, while FA treatment reduced their concentrations, indicating that FA can block the systemic inflammatory cascade induced by pyroptosis. GSDMD‐N and mature IL‐1β levels were elevated in MCAO/R mice, confirmed by Western blot, while GSDMD and pro‐IL‐1β levels showed no significant changes, suggesting that ischemia–reperfusion primarily induces the activation of pyroptosis effector proteins. FA intervention significantly declined GSDMD‐N and IL‐1β levels, indicating that its target is located in the downstream execution stage of the pyroptosis pathway (Figure 3M,N). These results indicate that FA reduces neuroinflammation after CIRI by hindering NLRP3 inflammasome activation and GSDMD‐induced pyroptosis in astrocytes.

### 3.4 FA Inhibits NLRP3 Inflammasome‐Mediated Astrocyte Pyroptosis via TLR4/NF‐κB Axis

**FIGURE 4:**
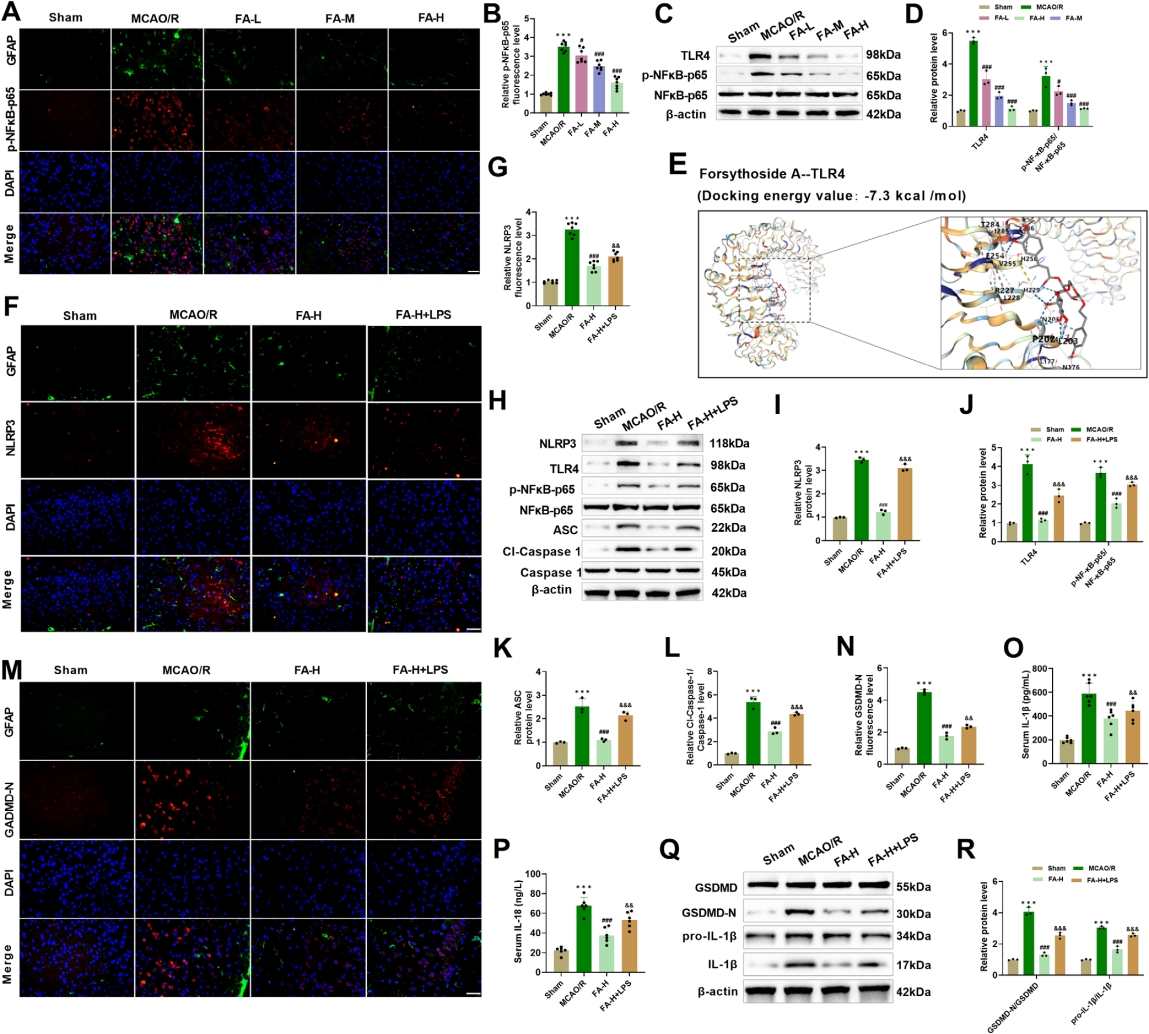
FA inhibits NLRP3 inflammasome‐induced astrocyte pyroptosis through regulating TLR4/NF‐κB. (A–B) Immunofluorescence detected colocalization of p‐NF‐κB‐p65 and GFAP in MCAO/R mice, and FA treatment reduced p‐NF‐κB‐p65 fluorescence intensity (40×, 50 μm). (C–D) Elevated TLR4 and p‐NF‐κB‐p65/NF‐κB‐p65 levels in MCAO/R mice were assessed through Western blot, and FA treatment reduced these protein levels. (E) Molecular docking results of TLR4 and FA. (F–G) Immunofluorescence measured the co‐localization of NLRP3 and GFAP in MCAO/R mice, and FA treatment declined NLRP3 level, whereas the addition of the TLR4 agonist, LPS, led to a rebound in NLRP3 levels (40×, 50 μm). (H–L) Western blot measured that FA treatment decreased TLR4, p‐NF‐κB‐p65/NF‐κB‐p65, NLRP3, Cl‐Caspase‐1/Caspase‐1, and ASC levels in MCAO/R mice, whereas co‐treatment with LPS led to a rebound in these protein levels. (M‐N) Immunofluorescence measured the co‐localization of GSDMD‐N and GFAP in MCAO/R mice, and FA decreased the fluorescence intensity of GSDMD‐N, whereas the addition of LPS led to a rebound in GSDMD‐N levels (40×, 50 μm). (O–P) ELISA results showed that FA treatment decreased serum IL‐18 and IL‐1β levels, but the addition of LPS resulted in increased levels of both. (Q–R) Western blot measured that FA treatment reduced GSDMD‐N and IL‐1β levels in MCAO/R mice, whereas LPS attenuated the effect of FA. ***p < 0.001 vs. Sham; *p < 0.05, **p < 0.01, ***p < 0.001 vs. MCAO/R; &&*p* < 0.01 vs. FA‐H + LPS.

To investigate the specific mechanism through which FA hinders NLRP3 inflammasome activation and astrocyte pyroptosis, we evaluated the related signaling pathways. In MCAO/R mice, p‐NF‐κB‐p65 and GFAP showed significant colocalization in ischemic penumbra, suggesting that NF‐κB was activated in activated astrocytes. After FA intervention, the fluorescence intensity of p‐NF‐κB‐p65 declined dose‐dependently, indicating that it could inhibit ischemia‐induced astrocyte NF‐κB nuclear translocation (Figure 4A,B). TLR4 and p‐NF‐κB‐p65/NF‐κB‐p65 levels were markedly elevated in MCAO/R mice, whereas FA treatment downregulated TLR4 and reduced NF‐κB p65 phosphorylation levels (Figure 4C,D). Furthermore, molecular docking studies revealed strong binding activities between FA and TLR4, with binding energies measured at −7.3 kcal/mol (Figure 4E). Immunofluorescence co‐localization of NLRP3 and GFAP showed that NLRP3 fluorescence intensity in astrocytes of MCAO/R mice was significantly elevated, while FA intervention could reduce its fluorescence intensity. It is worth noting that after treatment with the TLR4 agonist LPS, the fluorescence intensity of NLRP3 significantly increased again, implying that the suppressive impact of FA on NLRP3 may be influenced by TLR4 activity regulation (Figure 4F,G). In addition, FA treatment reduced TLR4, p‐NF‐κB‐p65, NLRP3, Cl‐Caspase‐1/Caspase‐1, and ASC protein levels in MCAO/R mice, while LPS treatment reversed the above effects, causing a rise in these proteins (Figure 4H–L). Colocalization analysis of GSDMD‐N and GFAP showed that FA intervention markedly weakened GSDMD‐N fluorescence intensity in astrocytes of MCAO/R mice, while LPS treatment reversed this effect, confirming that the suppressive impact of FA on pyroptosis depends on the TLR4 pathway (Figure 4M,N). FA treatment decreased the serum levels of IL‐18 and IL‐1β in MCAO/R mice, while LPS treatment caused a marked rise in their concentrations (Figure 4O,P). Moreover, FA declined GSDMD‐N and mature IL‐1β protein levels in MCAO/R mice, while GSDMD and pro‐IL‐1β showed no significant changes. LPS treatment notably weakened the suppressive effect of FA on GSDMD‐N and IL‐1β, further indicating that FA inhibits pyroptosis via the TLR4/NF‐κB axis (Figure 4Q,R). The above results confirmed that FA inhibited NLRP3 inflammasome activation via blocking the TLR4/NF‐κB axis, thus inhibiting astrocyte pyroptosis. The TLR4 agonist LPS further verified this conclusion.

### 3.5 Inhibitory Impact of FA on OGD/R‐Caused NLRP3 Inflammasome Activation in Astrocytes

**FIGURE 5:**
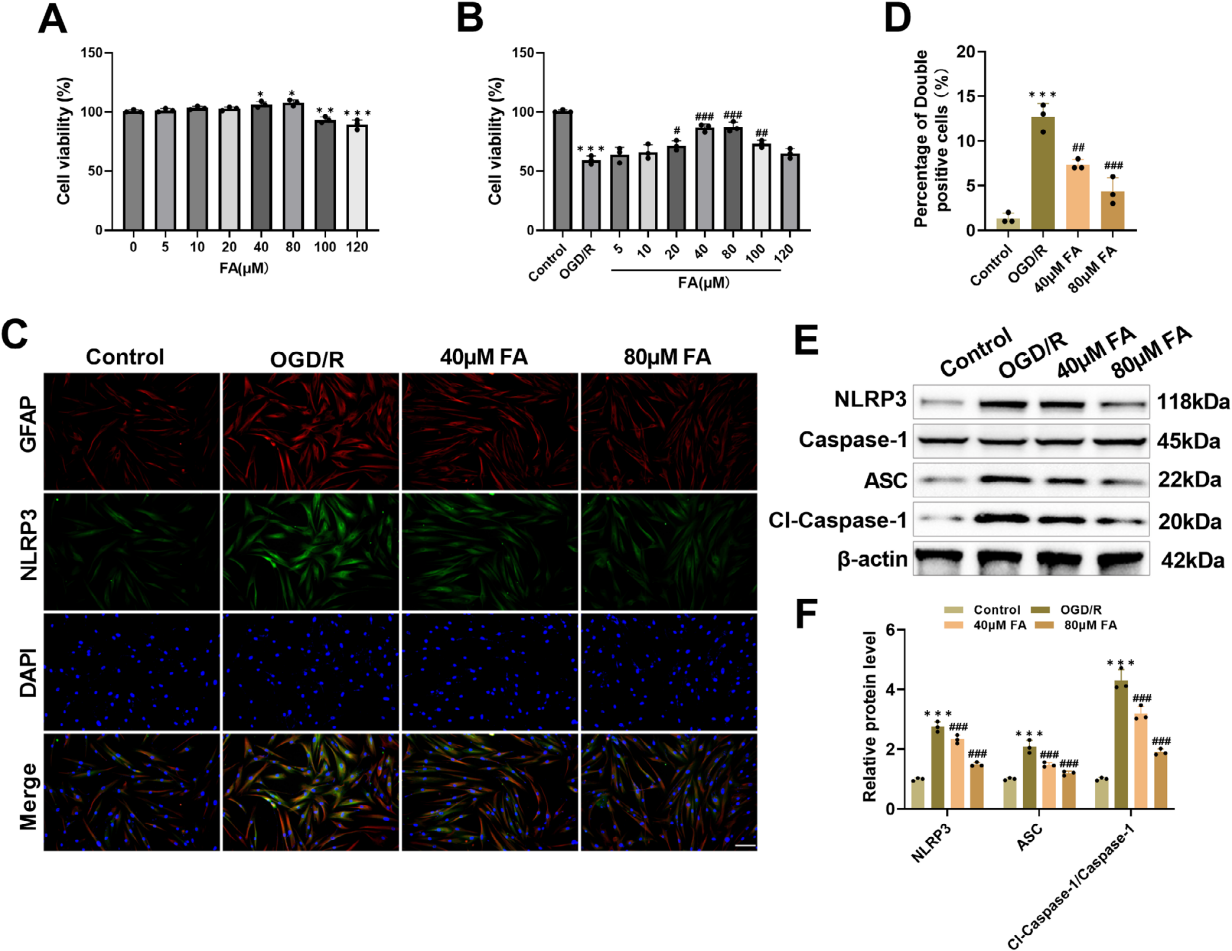
FA inhibits activation of NLRP3 inflammatory vesicles in OGD/R cells. (A) CCK‐8 assay detected the viability of U251 cells after 24 h of FA (5, 10, 20, 40, 80, 100, 120 μM) treatment. (B) CCK8 assay showed that FA (20–100 μM) treatment attenuated U251 cell viability impairment caused by OGD/R. (C–D) Immunofluorescence co‐localization measured that OGD/R resulted in increased fluorescence intensity of NLRP3 in U251 cells, and FA treatment decreased NLRP3 level (20×, 100 μm). (E–F) Western blot measured that OGD/R resulted in elevated NLRP3, Cl‐Caspase‐1/Caspase‐1, and ASC levels in U251 cells, whereas FA treatment decreased these proteins. *p < 0.05, **p < 0.01, ***p < 0.001 vs. Control; *p < 0.05, **p < 0.01, ***p < 0.001 vs. OGD/R.

Next, we used OGD/R‐induced U251 cells to investigate the beneficial effects of FA on neuroinflammation. Following treatment with FA for 24 h, the CCK‐8 assay showed that 5, 10, and 20 μM FA had no notable impact on cell viability, 40 and 80 μM FA markedly increased cell viability, and at 100 and 120 μM, cell viability declined considerably (Figure 5A). In the OGD/R cell model, 20–100 μM FA pretreatment significantly improved U251 cell viability damage (Figure 5B). Among them, 40 and 80 μM FA had the most significant effect on improving cell viability, so we used these two concentrations to evaluate the protective effect of FA. NLRP3 and GFAP showed obvious colocalization, and OGD/R caused a marked rise in NLRP3 fluorescence intensity. After FA treatment, NLRP3 fluorescence intensity was decreased markedly, confirming that it could suppress OGD/R‐induced NLRP3 expression (Figure 5C,D). In addition, OGD/R caused a notable rise in NLRP3, Cl‐Caspase‐1/Caspase‐1, and ASC levels in U251 cells, and FA intervention could dose‐dependently downregulate the above proteins (Figure 5E,F). These results indicate that FA can alleviate OGD/R‐induced astrocyte damage by hindering NLRP3 inflammasome assembly and activation.

### 3.6 FA Alleviates OGD/R‐Caused Astrocyte Pyroptosis and Inflammatory Response by Inhibiting NLRP3 Inflammasome Activation

**FIGURE 6:**
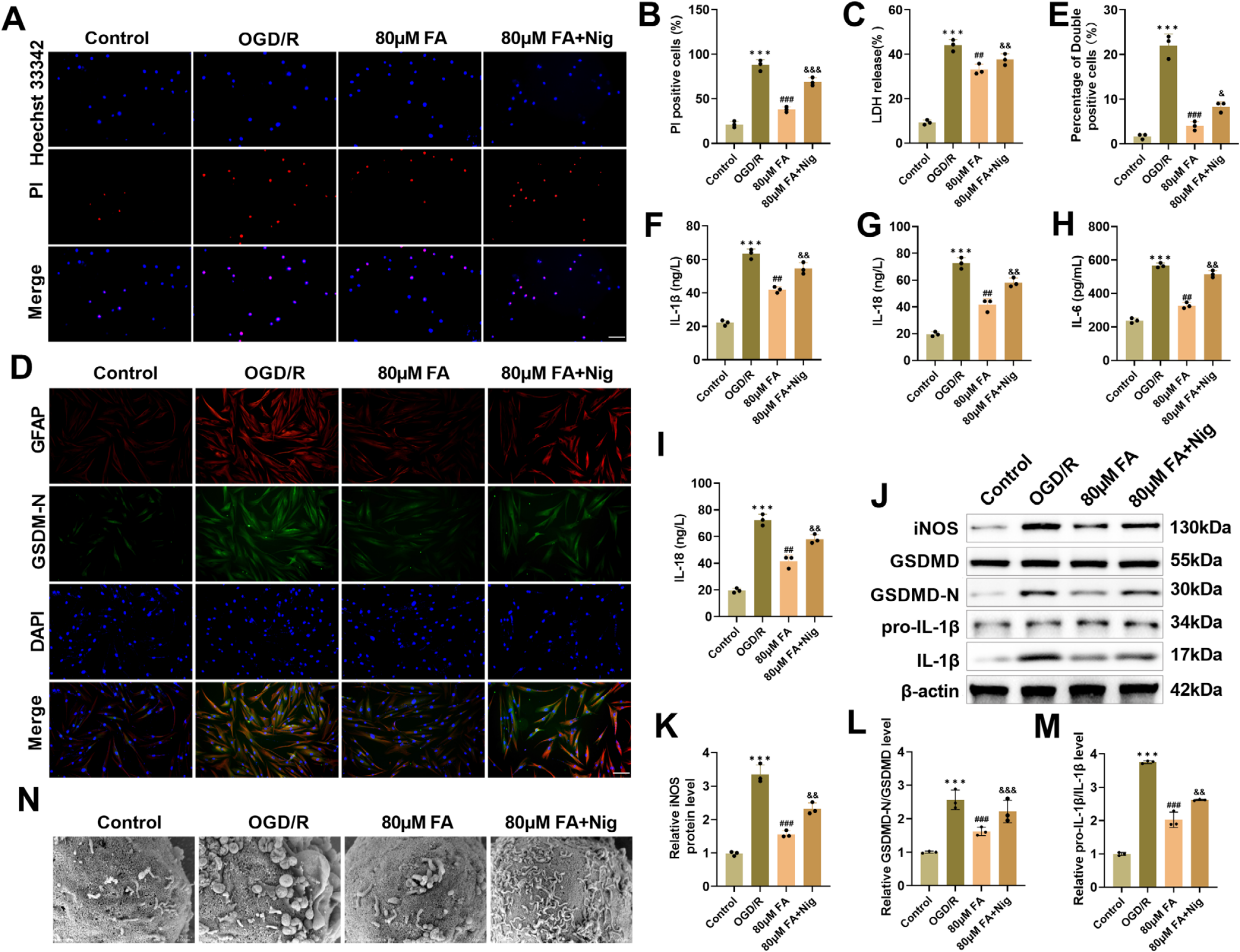
FA suppresses OGD/R astrocyte pyroptosis and inflammation through hindering NLRP3 inflammasome activation. (A–B) Hoechst 33,342 and PI staining showed that 80 μM FA reduced pyroptosis in U251 cells after OGD/R, whereas NLRP3 agonist Nig resulted in increased pyroptosis (20×, 100 μm). (C) LDH assay showed that LDH release from U251 cells was increased following OGD/R FA inhibited LDH release, while Nig led to a rebound in LDH release. (D–E) Immunofluorescence co‐localization measured that OGD/R resulted in increased GSDMD‐N fluorescence intensity in U251 cells, FA treatment decreased GSDMD‐N level, and Nig attenuated the effect of FA (20×, 100 μm). (F–I) ELISA indicated that OGD/R resulted in increased IL‐1β, TNF‐α, IL‐18, and IL‐6 levels; FA treatment declined these inflammatory factors, but addition of Nig resulted in increased levels. (J–M) Western blot measured that OGD/R caused elevated iNOS, IL‐1β, and GSDMD‐N levels in U251 cells, and FA treatment decreased these protein levels, whereas the addition of Nig caused a rebound in these protein levels. (N) SEM to observe the morphological changes associated with pyroptosis in U251 cells. ***p < 0.001 vs. Control; **p < 0.01 vs. OGD/R; &&*p* < 0.01 vs. 80 μM FA.

Hoechst 33,342/PI double staining results showed that after OGD/R treatment, U251 cells showed typical pyroptosis characteristics, nuclear condensation, and increased PI uptake, while FA (80 μM) markedly reduced the proportion of PI‐positive cells. Co‐treatment with the NLRP3 agonist Nig reversed this effect, causing a rise in pyroptotic cells, suggesting that FA exerts a protective impact on U251 cells through hindering NLRP3 inflammasome activation (Figure 6A,B). The cell membrane integrity was evaluated by detecting the amount of LDH released. OGD/R treatment markedly increased the LDH level in U251 cells, indicating that the cell membrane structure was damaged; while FA pretreatment reduced LDH release, indicating that it can effectively maintain the integrity of the cell membrane (Figure 6C). Immunofluorescence colocalization showed that GSDMD‐N fluorescence intensity in U251 cells was markedly enhanced after OGD/R. FA treatment reduced GSDMD‐N level, while cotreatment with Nig weakened the effect (Figure 6D,E). After OGD/R treatment, IL‐1β, TNF‐α, IL‐18, and IL‐6 levels in U251 cells were notably elevated. FA treatment decreased the concentrations of the above inflammatory factors, while co‐treatment with Nig resulted in a rise in these levels (Figure 6F–I). In addition, OGD/R led to increased iNOS, GSDMD‐N, and IL‐1β levels, while FA pretreatment markedly down‐regulated these proteins. Co‐treatment with Nig weakened the inhibitory impact of FA (Figure 6J–M). SEM observations showed that after OGD/R, U251 cells were obviously swollen, and holes appeared in the cell membrane, which was a typical scorched morphology. FA treatment could alleviate the above morphologic abnormalities, but Nig decreased the effect of FA (Figure 6N). The above results confirmed that FA alleviated OGD/R‐induced astrocyte pyroptosis and inflammatory response by inhibiting NLRP3 inflammasome activation.

### 3.7 FA Hinders OGD/R‐Caused NLRP3 Inflammasome Activation Trough TLR4/NF‐κB Axis

**FIGURE 7:**
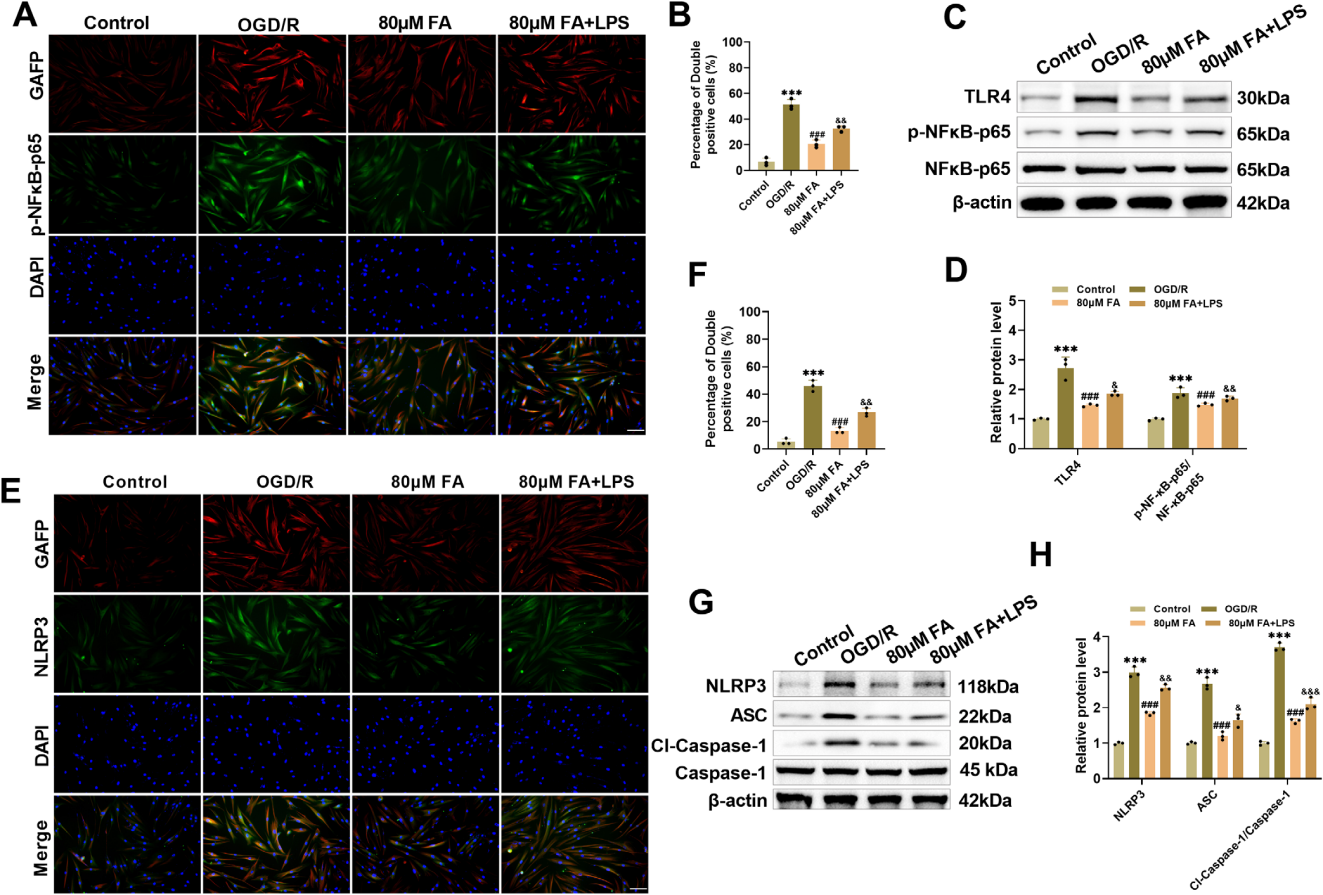
FA inhibits NLRP3 inflammasome activation through regulating TLR4/NF‐κB. (A–B) Immunofluorescence co‐localization measured that OGD/R resulted in increased p‐NF‐κB‐p65 fluorescence intensity in U251 cells, and FA treatment decreased p‐NF‐κB‐p65 levels, whereas the addition of LPS, the TLR4 agonist, resulted in a rebound of p‐NF‐κB‐p65 levels (20×, 100 μm). (C–D) OGD/R caused increased TLR4, p‐NF‐κB‐p65/NF‐κB‐p65 levels measured by Western blot, and FA treatment decreased the expression levels, while LPS weakened the inhibitory effect of FA. (E–F) Immunofluorescence co‐localization measured OGD/R led to increased NLRP3 fluorescence intensity in U251 cells, FA treatment decreased NLRP3 levels, and LPS led to a rebound in NLRP3 levels (20×, 100 μm). (G–H) OGD/R led to increased NLRP3, Cl‐Caspase 1/Caspase 1, and ASC levels revealed through Western blot, and FA decreased the levels of all three, whereas LPS weakened the inhibitory effect of FA. ***p < 0.001 vs. Control; *p < 0.05, **p < 0.01 vs. OGD/R; &&*p* < 0.01 vs. 80 μM FA.

Next, we investigated whether FA could also regulate the TLR4/NF‐κB pathway in vitro, thus inhibiting NLRP3 inflammasome activation. In U251 cells treated with OGD/R, p‐NF‐κB‐p65 colocalized with GFAP, implying that the NF‐κB was activated in activated astrocytes. FA treatment reduced p‐NF‐κB‐p65 fluorescence intensity, while co‐treatment with the TLR4 agonist LPS reversed this effect, resulting in a significant rise in p‐NF‐κB‐p65 fluorescence intensity (Figure 7A,B). After OGD/R, TLR4 and p‐NF‐κB‐p65 in U251 cells were elevated markedly, whereas FA treatment downregulated TLR4 expression and reduced NF‐κB p65 phosphorylation level; while LPS co‐treatment weakened the inhibitory effect of FA, resulting in a rebound in TLR4 and p‐NF‐κB‐p65 expression levels (Figure 7C,D). Immunofluorescence colocalization of NLRP3 and GFAP indicated that OGD/R treatment caused a notable rise in NLRP3 fluorescence intensity in U251 cells. FA pretreatment reduced NLRP3 fluorescence intensity, while LPS cotreatment led to a rebound in NLRP3 levels (Figure 7E,F). In addition, OGD/R caused a marked increase in NLRP3, Cl‐Caspase‐1/Caspase‐1, and ASC levels in U251 cells. FA pretreatment declined the levels of these proteins, and the addition of LPS reversed this effect (Figure 7G,H). These results confirm that FA inhibits NLRP3 inflammasome activation through blocking the TLR4/NF‐κB axis, thus blocking the pyroptosis cascade.

## 4 Discussion

CIRI‐mediated neuroinflammatory cascade and activation of neuronal death pathways are the key pathological mechanisms of ischemic stroke [9, 34]. Numerous studies have shown that Chinese herbal medicine has great prospects for treating ischemic stroke and cardiovascular and cerebrovascular diseases like coronary heart disease [35, 36, 37]. FA, an extract of *Forsythia suspensa* , has been demonstrated to alleviate brain edema and oxidative stress damage, and promote neurological function recovery in ischemic stroke rats [16]. However, whether it can inhibit neuroinflammation caused by CIRI and thereby inhibit neuronal pyroptosis remains unclear. Astrocytes are a key cell type involved in inflammatory response and neural repair in CIRI, and their activation and release of inflammatory factors (TNF‐α, IL‐6) through the TLR4/NF‐κB pathway is an important mechanism for exacerbating brain injury [38, 39]. Referring to Li et al. [22] and Xu et al. [26], we constructed an MCAO/R mouse model and an OGD/R U251 cell model to clarify the anti‐inflammatory and neuroprotective roles of FA in CIRI. We found that FA could decline cerebral infarction volume in mice after MCAO/R surgery, and alleviate the neurological deficit and pathological histological damage in mice. In addition, 40 and 80 μM FA were able to markedly increase the viability of U251 cells following OGD/R treatment, decrease LDH release, and had no adverse effects on normal U251 cells. These results all indicated that FA can improve neuronal damage, hinting at its possible therapeutic benefits for CIRI. In this study, the drug was administered 1 h after the completion of MCAO/R modeling. The early pathological events of CIRI (e.g., oxidative stress burst, initial activation of glial cells, and the beginning of BBB damage) mostly occur 0.5–2 h after reperfusion, and the intervention at this stage can minimize the conversion of primary injury to secondary injury, which makes it easy to specify the core target of drug action [9, 40]. In future studies, dosing at different time points is needed to explore the actual therapeutic window for FA.

Neuroinflammation caused by CIRI is a significant pathological mechanism that contributes to secondary brain injury [41]. Typically, BBB restricts the movement of immune cells from the periphery to the brain tissue [42]. However, after cerebral ischemia occurs, tissue hypoxia in the ischemic penumbra induces abnormal activation of astrocytes and microglia and recruits peripheral neutrophils and monocytes by releasing chemokines [43]. These immune cells infiltrate the brain tissue via the compromised BBB, triggering the release of inflammatory mediators, further inducing inflammatory responses and promoting neuronal damage and death [44]. Therefore, inhibiting neuroinflammation caused by CIRI may be an effective strategy for treating CIRI. FA has been shown to be effective in managing several inflammatory diseases such as colitis and diabetes [45, 46]. This study also found that FA effectively hindered the abnormal activation of astrocytes in MCAO/R mice and decreased the secretion of inflammatory cytokines in serum while reducing MPO activity and the number of apoptotic neurons. Based on the above results, FA may inhibit local and systemic inflammation in MCAO/R mice by inhibiting the abnormal activation of astrocytes. FA is the main active ingredient of Forsythiae Fructus, and its anti‐inflammatory effect is highly compatible with the traditional medicinal effect of Forsythiae Fructus, which is “clearing heat and detoxifying” [47]. In traditional Chinese medicine theory, the effect of “clearing heat and detoxifying” is mainly to relieve inflammatory symptoms caused by “internal heat and toxins” [48, 49]. In this study, FA inhibited neuroinflammation and reduced brain damage in MCAO model rats, which is a concrete manifestation of the traditional effect of *Forsythia suspensa* in “clearing heat and detoxifying” at the modern pharmacological level.

Pyroptosis is a type of pro‐inflammatory programmed cell death and is mediated by Caspase‐1 (classical pathway) or Caspase‐11/4/5 (non‐classical pathway), playing a key role in neuroinflammatory diseases [50]. At the molecular level, pyroptosis depends on the cleavage and activation of GSDMD. After NLRP3 recognizes damage‐associated molecular patterns (DAMPs), it brings in pro‐Caspase‐1 through ASC to assemble an inflammasome [51]. After that, pro‐Caspase‐1 is activated to generate mature Caspase‐1, which activates IL‐18 and IL‐1β on the one hand, and hydrolyzes the GSDMD protein on the other hand, producing GSDMD‐C and GSDMD‐N [52]. GSDMD‐N forms channels by binding to phospholipids in the cell membrane, causing osmotic imbalance and swelling and rupture of cells, and promoting the release of mature proinflammatory factors into the extracellular space, recruiting immune cells and amplifying local inflammatory responses [53, 54]. Pyroptotic activation of astrocytes can further exacerbate neuroinflammation in CIRI [55, 56]. Earlier studies have demonstrated that FA could hinder the activation of NLRP3 inflammasomes in mouse hippocampal tissue, suppress pyroptosis, and thereby alleviate brain damage induced by severe acute pancreatitis [24]. In our study, FA injection could inhibit NLRP3 inflammasome activation and inhibit neuronal pyroptosis in MCAO/R mice, aligning with the findings of Wang et al. [24]. The same occurrence was also noted in the OGD/R‐induced U251 cell model. Notably, NLRP3 agonists weakened the protective impact of FA on U251 cells, further confirming that FA alleviates cell pyroptosis by blocking the activation of NLRP3 inflammasomes, which may be an important mechanism for FA to exert neuroprotective effects.

**FIGURE 8:**
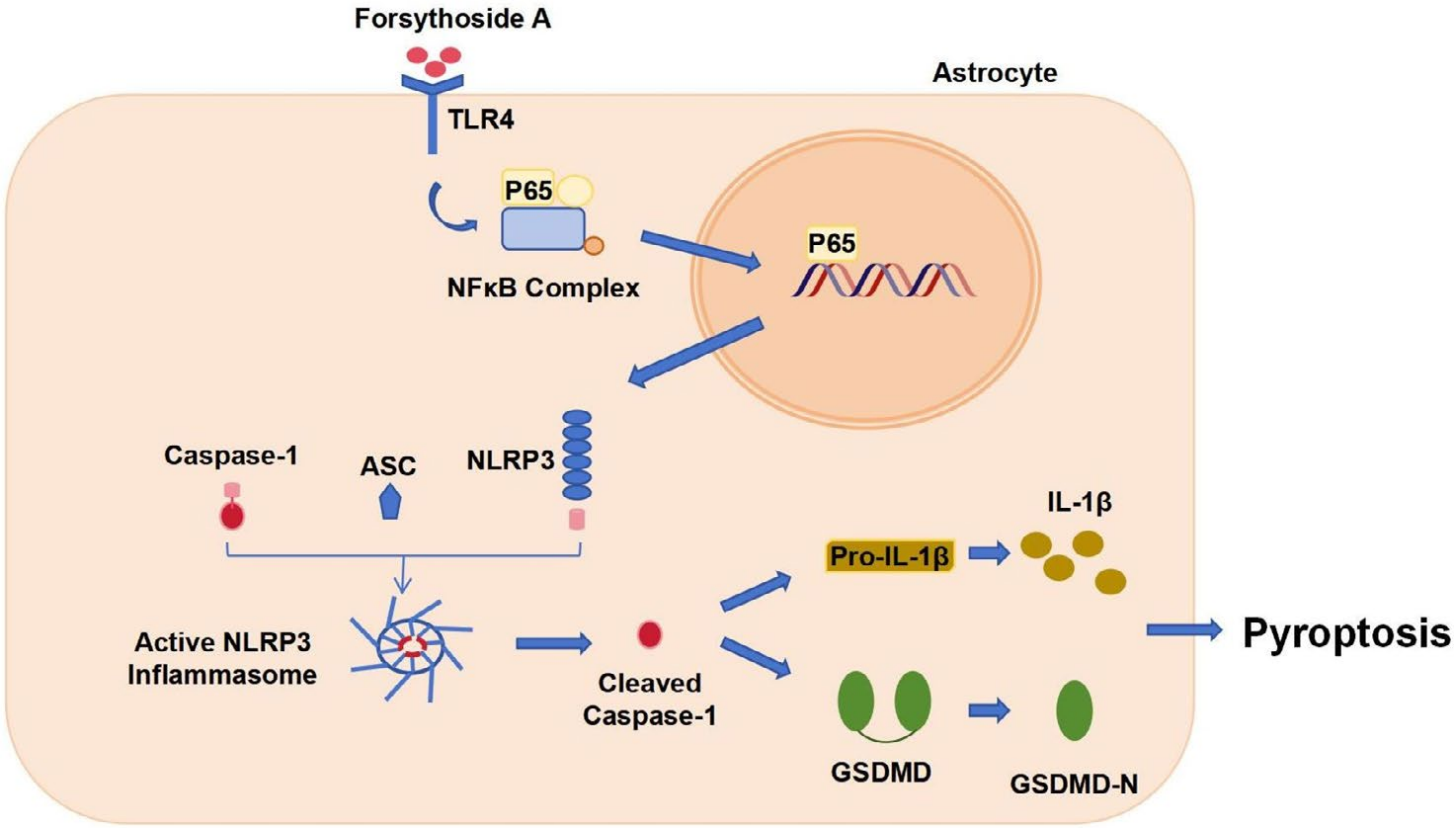
Mechanism of action. However, it needs to be recognized that there are still some limitations in this study. LPS is broad‐spectrum and can activate a variety of inflammation‐related signaling pathways. Subsequent studies can be validated using TLR4‐specific agonists to further confirm the specific regulatory relationship between FA and the TLR4 pathway. In addition, the mouse MCAO/R model and human U251 cells were used in this study, but the TLR4 signaling pathway may differ significantly between species and cell types, which could be a potential confounding factor, and future studies are needed to further validate the role of FA on the regulation of the TLR4 pathway between species and cells. In the MCAO/R model, only the sham surgery group was set up as a control, and no positive drug control group was included. Future studies can supplement the positive drug control experiments with Edaravone (a classic neuroprotective drug) to further verify the clinical application potential of FA and improve the clinical reference value of the research results. This study focused only on astrocyte apoptosis and did not explore the effect of FA on apoptosis and the function of other cell types (e.g., microglia, neurons) in CIRI. In future studies, the neuroprotective effects of FA in other cell types can be further explored to improve the network mapping of cellular mechanisms of FA effects. Furthermore, astrocyte‐specific knockout animal models (TLR4/pyroptosis‐related genes) can be used to further validate the cell‐specificity and pathway‐specificity of FA in regulating pyroptosis and exerting neuroprotective effects, to enhance the rigor and relevance of the study conclusions.

Toll‐like receptors can sense body damage and initiate pathway conduction, among which TLR4 is a key player in the process of CIRI [57, 58]. TLR4 activates the NF‐κB via MyD88, thus upregulating NLRP3 and participating in cell pyroptosis and inflammatory response [59, 60]. Cai et al. found that in MCAO/R rats and microglia injury models, inhibiting the TLR4/NF‐κB/NLRP3 axis reduced brain tissue damage, decreased cerebral infarction volume, and suppressed neuroinflammation [61]. Therefore, regulating the TLR4/NF‐κB/NLRP3 axis and preventing inflammatory infiltration of brain tissue cells and neuronal pyroptosis are effective ways to alleviate CIRI. We speculate that FA may inhibit NLRP3 inflammasome activation via blocking the TLR4/NF‐κB axis, thereby alleviating neuronal pyroptosis caused by CIRI. This study found that FA dose‐dependently downregulated TLR4 and inhibited NF‐κB p65 phosphorylation. Mechanistic research indicated that FA blocked NLRP3 inflammasome activation through a dual mechanism: On the one hand, FA inhibited TLR4 expression and NF‐κB p65 phosphorylation, weakening the transcriptional activation of NLRP3; on the other hand, FA reduced Caspase‐1 cleavage and GSDMD‐N generation, blocking the assembly of the inflammasome (Figure 8). Importantly, the suppressive impact of FA on NLRP3 inflammasome activation was reversed by the TLR4 agonist LPS, confirming that FA hinders NLRP3 inflammasome activation through regulating TLR4/NF‐κB, thereby alleviating neuronal pyroptosis in CIRI.

## 5 Conclusion

FA suppresses TLR4/NF‐κB axis activation, which inhibits NLRP3 inflammasome‐driven astrocyte pyroptosis, thus reducing the inflammatory cascade in the CIRI mouse model and cell model. This study revealed that FA inhibits neuroinflammation in CIRI via modulating the TLR4/NF‐κB/NLRP3 pathway, providing potential drugs and therapeutic targets for CIRI. In addition, the protective effect of FA in other neural cell types (such as microglia) can be explored in the future to further clarify the mechanism by which FA alleviates neuroinflammation in CIRI.

## Author Contributions

Yupeng Guo, Xuanwei Dong: Conceived and designed the research, conducted experiments, and analyzed data. Drafted and revised the manuscript critically for important intellectual content. Min Liu, Jianxin Wang: Contributed to the acquisition, analysis, and interpretation of data. Provided substantial intellectual input during the drafting and revision of the manuscript. Yupeng Guo, Shewei Guo: Participated in the conception and design of the study. Played a key role in data interpretation and manuscript preparation. All authors have read and approved the final version of the manuscript.

## Ethics Statement

This study was approved by the First Affiliated Hospital of Zhengzhou University Animal Ethics Committee (Approval No. 2021090201).

## Consent

The manuscript has neither been previously published nor is under consideration by any other journal. The authors have all approved the content of the paper.

## Conflicts of Interest

The authors declare no conflicts of interest.

## Data Availability

The data that support the findings of this study are available from the corresponding author upon reasonable request.
